# Review on the protective activity of osthole against the pathogenesis of osteoporosis

**DOI:** 10.3389/fphar.2023.1236893

**Published:** 2023-08-23

**Authors:** Jincai Chen, Xiaofei Liao, Juwen Gan

**Affiliations:** ^1^ Department of Orthopedics, First Affiliated Hospital of Gannan Medical University, Ganzhou, China; ^2^ Department of Pharmacy, Ganzhou People’s Hospital, Ganzhou, China; ^3^ Department of Pulmonary and Critical Care Medicine, Ganzhou People’s Hospital, Ganzhou, China

**Keywords:** osteoporosis, osthole, osteoblast, osteoclast, bone remodeling, Wnt/-catenin, BMP-2, RANKL

## Abstract

Osteoporosis (OP), characterized by continuous bone loss and increased fracture risk, has posed a challenge to patients and society. Long-term administration of current pharmacological agents may cause severe side effects. Traditional medicines, acting as alternative agents, show promise in treating OP. Osthole, a natural coumarin derivative separated from *Cnidium monnieri* (L.) Cusson and *Angelica pubescens* Maxim. f., exhibits protective effects against the pathological development of OP. Osthole increases osteoblast-related bone formation and decreases osteoclast-related bone resorption, suppressing OP-related fragility fracture. In addition, the metabolites of osthole may exhibit pharmacological effectiveness against OP development. Mechanically, osthole promotes osteogenic differentiation by activating the Wnt/β-catenin and BMP-2/Smad1/5/8 signaling pathways and suppresses RANKL-induced osteoclastogenesis and osteoclast activity. Thus, osthole may become a promising agent to protect against OP development. However, more studies should be performed due to, at least in part, the uncertainty of drug targets. Further pharmacological investigation of osthole in OP treatment might lead to the development of potential drug candidates.

## Graphical Abstract

**Figure:**
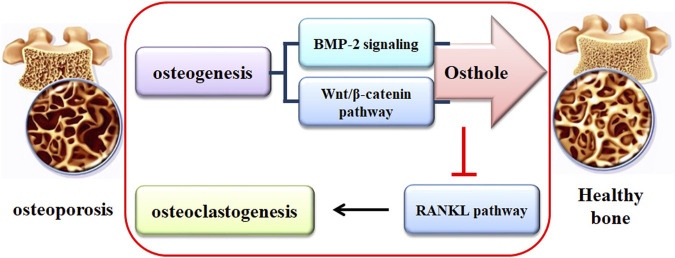

## 1 Introduction

Osteoporosis (OP) is characterized by the gradual decline of bone mass and the deterioration of bone microstructure. Osteoporotic bone is fragile, and the fracture risk in patients with OP is enhanced. OP has been defined as a “progressive systemic skeletal disease” by the World Health Organization (WHO) and become a public health concern worldwide (Compston et al., 2017). The clinical data indicate that the number of OP patients with fractures is increasing. In the UK, about 536,000 new patients with fragility fractures appear annually. In 2010, approximately 22,000,000 women and 5,500,000 men had OP in the European Union, and the costs for the incidents and prior fragility fractures are estimated at € 37 billion (Svedbom et al., 2013). It has been reported that more than 1/3 of adult women and 1/5 of adult men may experience one or more fragility fractures in their lifetime (van Staa et al., 2001). OP and the fragility fracture have posed challenges to individuals and society. Thus, an in-depth understanding of OP development is essential because it may provide the scientific management for OP prevention and treatment.

Pharmacological treatment for OP includes anti-resorptive agents, such as bisphosphonates, selective estrogen receptor modulators (SERMs), calcitonin, and denosumab, and anabolic drugs, such as abaloparatide, romosozumab, and teriparatide. However, the side effects of these pharmacological drugs have been reported (Kim et al., 2021). For example, the common adverse effects of bisphosphonates by oral administration are dysphagia, abdominal pain, nausea, flatulence, constipation or diarrhea, acid regurgitation, taste distortion, esophageal ulcers, and gastritis (Schuiling et al., 2011; Qaseem et al., 2017; Akkawi and Zmerly, 2018). SERMs can increase the risk of stroke, thromboembolic disorders, and muscle cramps (Rochefort, 2014; Kim et al., 2017). Calcitonin also causes adverse effects, such as hypocalcemia, nasal adverse reactions, formation of calcitonin antibodies, and prostate cancer (Tabatabaei-Malazy et al., 2017; Akkawi and Zmerly, 2018). Long-term administration of denosumab can cause osteonecrosis of the jaw (ONJ), atypical femoral fractures (AFFs), hypocalcemia, musculoskeletal pain, and gastrointestinal symptoms (Qaseem et al., 2017; Tabatabaei-Malazy et al., 2017; Akkawi and Zmerly, 2018). The side effects of abaloparatide and teriparatide include dizziness, headache, nausea, and leg cramps (Fukumoto and Matsumoto, 2017). The subcutaneous injection of romosozumab may induce stroke, cardiovascular events, and myocardial infarction (Rachner et al., 2019). Combinational therapy of anti-resorptive and anabolic drugs is considered the ideal protocol for OP treatment. However, studies have shown that combinational therapy only slightly increases BMD, and it is not recommended for OP treatment due to the combined side effects (Martiniakova et al., 2020; Kim et al., 2021). Thus, it is essential to explore an alternative strategy for OP treatment.

**FIGURE 1:**
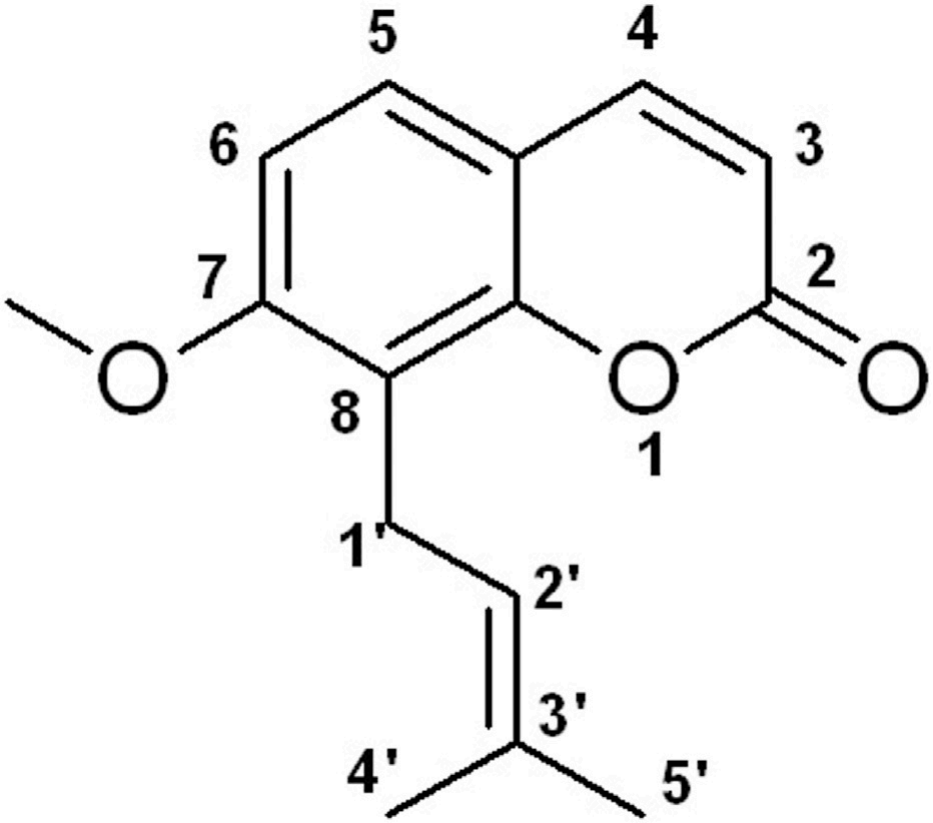
The chemical structure of osthole.

Natural medicines exhibit promise in preventing and treating various human diseases due to their long history of use, pharmacological effects, and limited side effects (AbouAitah and Lojkowski, 2021; Nannan et al., 2023). Osthole, 7-methoxy-8-(3-methyl-2-butenyl) coumarin (Figure 1), is a natural coumarin derivative separated from several medicinal plants, such as *Cnidium monnieri* (L.) Cusson and *Angelica pubescens* Maxim. f. A study has shown that osthole exhibits various pharmacological activities, such as anti-inflammation, anti-tumor, liver protection, neuroprotection, immunomodulator, and bone protection (Zhang et al., 2015). Interestingly, osthole has been reported to inhibit osteoclast formation and function, promote osteoblast differentiation and bone formation, increase bone mineral density (BMD) and bone strength, and enhance fracture healing (Sun et al., 2021; Wu et al., 2022). Osthole may become a potential agent for OP treatment. In this article, we will discuss the effects of osthole on bone remodeling.

The literature review focuses on English-written books and articles that are published in Pubmed, Scopus, and Medline databases. The search terms, such as osthole, osteoporosis, osteogenic differentiation, osteoblast, osteoclast, and osteoclastogenesis, were used. The search results in the databases were checked to obtain informative articles.

## 2 The pathogenesis of OP

**FIGURE 2:**
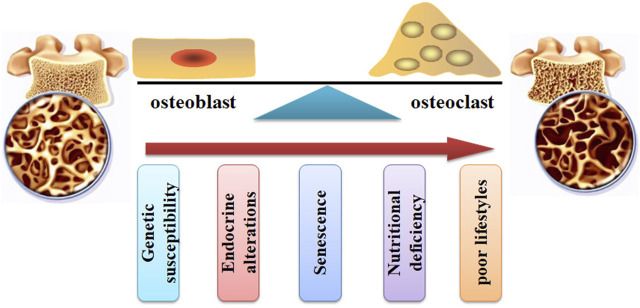
The pathogenesis of OP. The bone remodeling can be interrupted by increased osteoclast activity and decreased osteoblast function. Many factors, such as genetic susceptibility, endocrine alterations, senescence, nutritional deficiency, and poor lifestyles, can stimulate the pathological development of OP.

Bone, a dynamic tissue, is orchestrated by a balance between formation and resorption (Figure 2). Bone remodeling is the process of replacing old bones with new bones. The balance between osteoblast-mediated new bone formation and osteoclast-controlled bone resorption is controlled by physical and hormonal factors (Tella and Gallagher, 2014). During the pathogenesis of OP, the balance is interrupted, and the activities of osteoblasts and osteoclasts are dysregulated. The homeostasis of bone remodeling is interrupted continuously. Subsequently, bone mass loss and bone micro-architecture damage are deteriorated. Specifically, increased osteoclast activity and decreased osteoblast function may result in bone loss and skeletal fragility (Kamel, 2006).

Many factors (Figure 2), such as genetic susceptibility, endocrine alterations, senescence, nutritional deficiency, and poor lifestyles, have been associated with an increased risk of bone and fragility fractures (Wang et al., 2023b; Valenzuela-Martínez et al., 2023). For example, aging and age-related disorders may cause an elevated level of pro-inflammatory cytokines, such as IL-6, IL-1β, and TNFα, which contribute to the imbalance of bone homeostasis (Di Munno and Ferro, 2019). Menopause and senescence can interrupt the balance between osteoblast-related bone formation and osteoclast-related bone resorption, disturbing bone turnover (Zhu and Zheng, 2021). Glucocorticoid (GC) treatment can induce OP development and fragility fractures. It has been reported that GCs stimulate the development of OP by decreasing osteoprotegerin expression and increasing receptor activator of nuclear factor κB ligand (RANKL) expression, leading to bone resorption enhancement, BMD reduction, and increased fracture risk (Compston, 2018).

Calcium, phosphorus, and magnesium are important components of bone, and the alterations of these components may result in an imbalance between bone synthesis and bone loss (Rizzoli et al., 2021). Calcium and vitamin D insufficiency can increase the risk of OP and fragility fractures (Polzonetti et al., 2020). In addition, inadequate intake of calcium and vitamin D also attenuates the efficacy of anti-OP drugs (Bonjour et al., 2009). Bone metabolism is mediated by several hormones, such as thyroid hormones, parathyroid hormone (PTH), estrogen, and testosterone. Hyperthyroidism induced by thyroid-stimulating hormone-suppressive therapy in patients with thyroid cancer contributes to OP development and the risk of fractures (Hong and Kang, 2023). Chronic PTH deficiency may induce bone microstructure alterations, bone remodeling imbalance, and increased fragility fractures (Ahn et al., 2023). In addition, continuous exposure to high levels of PTH reduces BMD and increases the risks of fragility fractures by increasing bone resorption (Silva and Bilezikian, 2021). It has been reported that a combination of calcitonin with PTH may maintain the balance between calcium and phosphorus, promoting bone metabolism (Matikainen et al., 2021). Pro- and anti-inflammatory cytokines mediate of osteoclast and osteoblast activity during inflammatory bone destruction. High levels of pro-inflammatory cytokines, such as IL-1β, IL-6, TNFα, and RANKL, are found in the peri-implant crevicular fluid, which contributes to bone loss (Berglundh et al., 2018). Chronic inflammation suppresses osteoblast differentiation and stimulates osteoclast activity, resulting in the reduction of bone formation and the induction of bone resorption (Wei et al., 2022). Cytokines, such as TNFα and RANKL, are indispensable for osteoclast differentiation. However, Cytokines in the immune system, such as IL-4 and IL-10, limit osteoclastogenesis and bone resorption. Cytokine-mediated immunomodulation in osteoclasts may be a potential strategy for OP treatment (Zhou et al., 2022).

OP can be primary and secondary. Primary OP, such as post-menopausal OP and senile OP, is often associated with aging and likely occurs in the elderly people. Secondary OP is related to some complications induced by diseases or drugs. Post-menopausal OP highlights the role of estrogen in bone metabolism. Menopausal women, due to estrogen deficiency, have a high rate of OP and fragility fracture. Estrogen interacts with the estrogen receptors (ERs) in the cytoplasm. Then, ERs dimerize and translocate to the nucleus for transcriptional regulation of target genes by binding to the specific DNA sequences, known as estrogen response elements (EREs) (Almeida et al., 2017). ERα and ERβ are the two types of ERs, and they exhibit different affinities to estrogen and interact with different DNA-binding domains. Interestingly, the SERMs have been developed for the therapeutic management of OP (Oh et al., 2022).

Estrogen has been reported to promote osteoblast survival, stimulate autophagy and reduce apoptosis. In addition, estrogen also increases osteoblast function by activating the bone morphogenetic protein 2 (BMP-2)/Smad1/5/8 and Wnt/β-catenin signaling pathways (Almeida et al., 2013). Estrogen may block RANKL/M-CSF-mediated activator protein-1 (AP-1)-dependent transcription, suppressing RANKL-induced osteoclast differentiation. It is reported that ERα can interact with BCAR1 and increase its activity, inhibiting RANKL/NF-κB-mediated osteoclast differentiation (Robinson et al., 2009). Senile OP is related to the senescent alterations of osteoblasts and osteocytes. Senescence is a complex process and affects various biological activities at the molecular and cellular levels. Mesenchymal stem cells (MSCs), a group of progenitor cells derived from bone marrow, can be orientated to osteogenic and adipogenic differentiation. However, it has been reported that an age-associated lineage switch from osteogenic to adipogenic differentiation in the senescent bone marrow mesenchymal stem cells (BMSCs) has been observed (Li et al., 2018). In addition, senescence may impair the differentiation, proliferation, lifespan, and function of osteoblasts, and thus affecting bone formation (Farr et al., 2017).

## 3 The protective activity of osthole against the pathogenesis of OP

Osthole, at doses of 40–320 μg/mL, has been reported to promote osteoblast proliferation and differentiation and induce new bone formation (Zhang et al., 2010). In ovariectomized (OVX)-induced rat OP (Table 1), osthole at the dose of 9 mg/kg by oral administration (5 days/week for 4 weeks) has been reported to prevent cancellous bone loss owing to estrogen deficiency in the femoral neck (Li et al., 2002). It has been shown that osthole can promote the proliferation and alkaline phosphatase (ALP) activity of Saos-2 cells by increasing the phosphorylation of Akt, ERK, and p38 (Table 2). However, treatment with an estrogen receptor antagonist ICI182,780 may abolish the effects of osthole on Saos-2 cells, indicating the potential estrogenic and anti-osteoporotic activities (Jia et al., 2016). In addition, ovariectomy in rats can increase the weight of bone and the levels of IL-6, TNFα, and Ca^2+^ and decrease BMD and the expression of TGFβ1, NO, and NOS. These abnormal alterations can be improved by osthole treatment (Si et al., 2020). Another study reports that osthole (5 mg/kg/day) can significantly increase BMD, improve biomechanical properties, promote new bone formation, and inhibit bone loss (Tang et al., 2010) (Table 1). Osthole is the main active compound in the chloroform fraction from the alcoholic extract of the fruits of *Cnidium monnieri* (L.) Cuss. The extracts have exhibited stimulatory effects on the proliferation of the osteoblast-like UMR106 cells (Meng et al., 2004).

**TABLE 1:** The protective activity of osthole against OP development in animal models.

Models	Doses	Routines	Biological actions	Efficacy	Ref
OVX-treated rats	9 mg/kg/day, 5 days/week, 4 weeks	Oral gavage	Cancellous bone mass↑, serum osteocalcin↑, urinary deoxypyridinoline↓	Osthole can be as effective as 17β-estradiol in suppressing bone loss	Li et al. (2002)
OVX-treated rats	400 mg/kg/day for 8 weeks	Oral gavage	BMD↑, IL-6↓, TNFα↓, Ca^2+^↓, Mg^2+^↓, P^2-^↑, NO↑, NOS↑, alkaline phosphatase↓, osteocalcin↑	Osthole inhibits bone resorption and accelerates bone formation	Si et al. (2020)
ICR Swiss mice	1 and 5 mg/kg, twice/day for 5 days	Subcutaneous injection	Bone formation↑, mineral appositional rate↑, bone-formation rate↑	Osthole increases mineral appositional rate and bone-formation rate	Tang et al. (2010)
OVX-treated rats	100 mg/kg/day for 8 weeks	Intraperitoneal injection	BMD↑, BV/TV↑, Tb.Th↑, Tb.Sp↓	Osthole increases total BMD, trabecular bone volume, and trabecular thickness, decrease trabecular separation, and improves biomechanical properties	
*BMP2* ^*ColCre*^ mice	30 mg/kg/day for 28 days	Subcutaneous injection	fracture gaps↓, bone volume↑, bone mechanical strength↑, the maximum torque and stiffness↑, Alcian blue staining↑, Sox9↑, Col2a1↑, Col10a1↑, Runx2↑, osteocalcin↑, p-Smad1/5/8↑	Osthole promotes bone strength and enhances fracture healing by activating the BMP2 signaling	Wang et al. (2017)
A femoral open fracture mouse model	5, 20, and 50 mg/kg/day for 4 weeks	Oral gavage	The callus area↓, BV/TV↑, TMD↑, BMD↑, mineralization rate↑, BMP-2↑, ALP↑, OCN↑, Col-X↑, and Col-I↑	Osthole promotes endochondral ossification by up-regulating maturation osteogenic marker gene expression and enhancing fracture repair and bony fusion	Zhang et al. (2016)
OVX-treated mice	10 mg/kg/day for 3 months	Intragastrical administration	Bone loss↓, Tb.N↑, Tb.Sp↓, TRAP-positive cells↓, NFATc1↓	Osthole inhibits RANKL-mediated osteoclastogenesis and prevents bone loss	Zhao et al. (2018)
12-month-old mice	5 mg/kg/day for 4 weeks	Intraperitoneal injection	Bone loss↓, BV/TV↑, Tb.N↑, Tb.Sp↓, TRAP-positive cells↓	Osthole inhibits osteoclast formation	Jin et al. (2021)
5/6-NCT mice	5 mg/kg/day for 2 months	Intraperitoneal injection	BMD↑, BV/TV↑, Tb.Th↑, TRAP-positive cells↓, TRAP↓, Cathepsin K↓, MMP-9↓, OPG↑, OPG/RANKL↑	Osthole inhibits osteoclast formation and partially reverses 5/6-NCT-induced bone loss by activating the OPG/RANKL signaling	Li et al. (2016)
TCP particle -implanted mice	10 mg/kg/day for 2 weeks	Subcutaneous injection into the calvaria	calvaria weight↑, osteoclast number↓, osteolysis area↓, serum osteocalcin↑, ALP↑, TRAP↓, cathepsin K↓, GRP78↓, CHOP↓, IL-6↓, TNFα↓	Osthole inhibits TCP particle-induced osteolysis by suppressing ER stress	Lv et al. (2016)
Thiram-treated chicken	20 mg/kg for 18 days	Oral gavage	ALP↑, SOD↑, T-AOC↑, GSH-Px↑, AST↓, ALT↓, MDA↓, BMP-2↑, Runx-2↑	Osthole inhibits oxidative stress and suppresses tibial dyschondroplasia	Waqas et al. (2019)

**TABLE 2:** The protective activity of osthole against OP development *in vitro*.

Cell lines	Concentrations	Biological activities	Ref
Rat osteoblasts	40, 80, 160, and 320 μg/mL	Cell proliferation↑, ALP↑, and nodule number↑	Zhang et al. (2010)
Saos-2 cells	1 μmol/L	Cell proliferation↑, ALP↑, p-p38↑, p-AKT↑, p-ERK1/2↑	Jia et al. (2016)
Primary mouse calvarial osteoblasts	10, 50, and 100 μM	Col-I↑, BSP↑, osteocalcin↑, ALP↑, nodule formation↑, BMP-2↑, p-Smad1/5/8↑, Wnt1↑, Wnt3a↑, Wnt4↑, and *β*-catenin↑	Tang et al. (2010)
BMMs derived from OVX mice	5 μM	c-Fos↓, NFATc1↓, MMP9↓, Ctsk↓, TRAP↓, no effect on the activation of the IκBα, JNK, ERK, or p38 pathways	Zhao et al. (2018)
RANKL-treated RAW264.7 cells	10^−6^ and 10^−5^ mol/L	TRAP-positive cells↓, CTSK↓, c-Src↓, β3-integrin↓, MMP-9↓, NFATc1↓, TRAP↓, p-IκBα↓, p-p65↓	Ma et al. (2019)
BMSCs derived from *OPG* ^*−/−*^ mice	100 μM	OPG↑, *β*-catenin↑	Jin et al. (2021)
Primary rat osteoblasts	10^−5^ mol/L	ALP↑, osteocalcin↑, calcium sediment↑, bFGF↑, IGF-I↑, Runx-2↑, BMP-2↑, osterix↑, p38 MAPK↑, Col-I↑	Ming et al. (2011)
MG-63 and hFOB cells	1, 5, 10, and 20 μM	ALP↑, osteocalcin↑, osteopontin↑, Col-I↑, BMP-2↑, p-Smad1/5/8↑, p-ERK1/2↑, p-p38↑	Kuo et al. (2005)
MC3T3-E1 cells	20, 50, and 100 μM	ALP↑, mineralization↑, BMP-2↑, OCN↑, Col-1↑, Runx-2↑, cAMP↑, p-CREB↑, osterix↑	Zhang et al. (2017)
LPS- and RANKL-treated BMMs	6.25, 12.5, and 25 μM	ROS↓, iNOS↓, CD206↑, CD86↓, CCR-7↓, IL-6↓, IL-10↑, NFATc1↓, TRAP↓, DC-STAMP↓, CTSK↓, c-Fos↓, V-ATPase D2↓, CTR↓, p-p65/p65↓, p-p38/p-38↓	Wang et al. (2023a)

**FIGURE 3:**
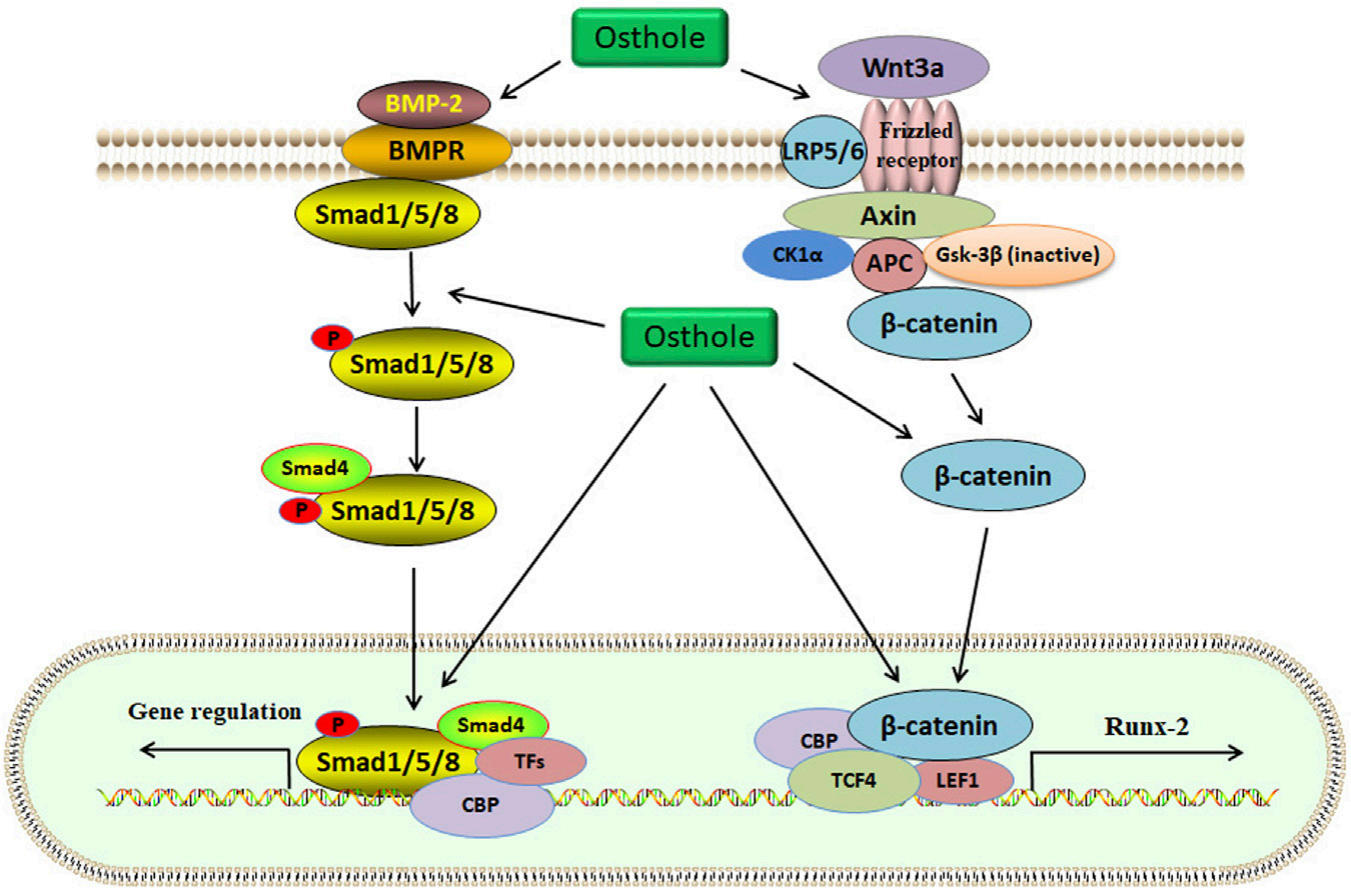
The Wnt/β-catenin and BMP-2/Smad1/5/8 signaling pathways are activated by osthole in osteoblasts. Wnt3a binds to the Frizzled receptor, and GSK-3β is inactivated. *β*-catenin becomes stable and accumulates in the cytoplasm. Then, *β*-catenin is translocated into the nucleus for transcriptional regulation of target genes, such as Runx-2. Osthole promotes the binding of wnt3a to the receptor, induces the stability of *β*-catenin, and stimulates *β*-catenin-mediated transcription. BMP-2 activates Smad1/5/8 by interacting with BMPR. Smad1/5/8 and Smad4 form a complex, which regulates target gene expression in the nucleus. Osthole activates BMP-2, induces Smad1/5/8 phosphorylation, and promotes the expression of target genes.

The clinical applications of bisphosphonates have received satisfactory results during OP treatment. However, their long-term effects on patients with fragility fractures are still controversial, due to the number reduction of both osteoclasts and osteoblasts and the higher risk of atypical femur fractures (Sellmeyer, 2010). The beneficial roles of osthole in promoting bone fracture healing have been confirmed in mouse models. Specifically, osthole (30 mg/kg/day for 28 days by local subcutaneous injection) increases the expression of the early cartilage markers Col2a1 and Sox9 and enlarges the cartilage area on day 7 (Table 1). Upregulated expression of Smad1/5/8 in chondrocytes is associated with chondrocyte maturation, which is a crucial step in endochondral ossification. Osthole increases the phosphorylation of Smad1/5/8 and promotes nuclear translocation of phosphorylated Smad1/5/8 after the recruitment of Smad4 on day 7, suggesting activation of the BMP-2 signaling (Figure 3). In addition, osthole upregulates the expression of the later cartilage hypertrophic marker Col10a1 on day 10 and promotes the expression of bone markers Runx-2 and OCN on day 21, stimulating endochondral ossification. In *BMP2* ^*ColCre*^ mice, the bone volume, the mechanical strength, and the stiffness of the tibia are significantly decreased, indicating the delayed progress of bone fracture healing (Wang et al., 2017). Consistently, gavage administration of osthole (20 mg/kg/day) increases BMD, promotes bone callus formation, and increases endochondral ossification at the end of week 2 after operation in mice. In addition, the immunohistochemical examination shows that the expression of BMP-2, ALP, OCN, Col-X, and Col-I is significantly elevated (Zhang et al., 2016) (Table 1).

## 4 Osthole promotes osteogenic differentiation

BMSCs, the predominant precursor cells of osteoblasts, contribute to bone regeneration and remodeling. In rat BMSCs, osthole (10^−5^ M) upregulates the expression of ALP, Col-I, BMP-2, osteocalcin, osteopontin, insulin-like growth factor-1 (IGF-1), runt-related gene 2 (Runx-2), osterix, and OPG, increases the ratio of OPG/RANKL, enhances the formation of mineralized nodules, and promotes the proliferation and osteogenic differentiation, stimulating new bone formation and inhibiting bone resorption (Zhai et al., 2014). Consistently, osthole (0–100 μM) increases the ALP activity and calcium nodule formation in a dose-dependent manner in MC3T3-E1 cells. Osthole promotes osteogenesis and increases bone growth and strength, as shown by increased expression of BMP-2, Runx-2, Osx, OCN, and Col-1 (Table 2). A BMP-2 inhibitor noggin can completely abolish osthole-activated osteogenesis (Zhang et al., 2017). The binding of the ligand Wnt3a to the frizzled receptor and co-receptor low-density lipoprotein receptor-related protein 5/6 (LRP5/6) may induce the phosphorylation of LRP5/6, which recruits Dishevelled, Axin, CK1α, and APC to form a complex. Gsk-3β will be inactivated, and *β*-catenin is stabilized. Then, *β*-catenin enters the nucleus and forms a complex with CBP, TCF4, and LEF1 to regulate the expression of target genes, such as Runx-2 (Dan et al., 2023). Ethanol has been reported to impair the differentiation of BMSCs into osteoblasts and alter the homeostasis of bone. Osthole (10, 50, and 100 μM) can improve ethanol-inhibited ALP, Col-I, OCN, and OPN expression and promote the proliferation and osteogenic differentiation of BMSCs by activating the Wnt/β-catenin signaling (Figure 3). Wnt antagonist JW74 can abolish the effects of osthole on BMSCs. In addition, osthole (10, 50, and 100 μM) also upregulates the expression of vascular endothelial growth factor (VEGF) and rescues ethanol-impaired tube formation in human umbilical vein endothelial cells (HUVECs) (Yu et al., 2020).

Osteoblasts are crucial for new bone formation and bone density. During osteoblast differentiation, the accumulation of collagenous matrix appears. Then, the expression of ALP is upregulated, and the secretion of osteocalcin is stimulated. Finally, the mineralization of bone nodules is formed. Some factors, such as Runx-2, BMP-2, p38 mitogen-activated protein kinase (p38-MAPK), and osterix, participate in the processes of new bone formation (Jeon et al., 2006). Osthole (10^−5^ mol/L) has been reported to induce new bone formation by stimulating ALP activity, increasing osteocalcin production, and promoting calcium sediment and mineralization of bone nodules (Ming et al., 2011). Patients with OP fracture often show decreased activity in human skeletal muscle satellite cells (hSMSCs) proliferation and osteogenic differentiation. Osthole (10^−6^ mol/L) has been reported to enhance cell proliferation, increase the expression of ALP, Col-I, Runx-2, and BMP-2, and promote the migration of hSMSCs to the fracture sites for repairment by up-regulating the expression of *β*-catenin. Conditional knockout of *β*-catenin SMSCs in *Pax7-Cre* ^*ERT2/+*^ *;β-catenin* ^*flox/flox*^ mice induces the delayed healing of tibial fractures (Jin et al., 2022).

MSCs with multipotent differentiation potentials have been applied to treat various diseases. Activation of osteoblast differentiation and inhibition of adipocyte differentiation of BMSCs may alleviate the development of OP. It has been reported that allogeneic MSCs injection may stimulate fracture healing (Huang et al., 2015). Autophagy plays a critical role in bone metabolism. It has been reported that knockout of Atg7 in osteoblasts can induce a decrease in trabecular bone mass and bone formation (Piemontese et al., 2016). It has been reported that osthole-treated BMMSCs exhibit better biomechanical properties than BMSCs when they are intravenously injected into the OVX-treated mice. In addition, osthole can enhance the serum levels of P1NP, ALP, and calcium and decrease that of TRAP by stimulating autophagy, as shown by increased expression of Beclin 1 and LC3II/I (Zheng et al., 2019). BMSCs are positively affected by the microenvironment, and their immunoregulatory characteristics can be affected. It has been reported that BMSCs isolated from patients with OP are much ineffective in cell therapy, and this might be associated with the declined capacity of osteoporotic BMSCs in inducing T-cell apoptosis due to the decreased protein expression of Fas and FasL (Liao et al., 2018). Osthole has immunomodulatory activity on BMSCs derived from OP mice. Specifically, osthole restores BMSC-mediated migration of T-cells by up-regulating the expression of MCP-1, which is a critical chemokine. In addition, the apoptosis of T-cells in the osthole-treated BMSCs group is increased. The protein expression of Fas and FasL is also upregulated. Mechanically, osthole-pretreatment may rescue the immunosuppressive activity of BMSCs, as shown by decreased expression of IL-6, IL-1β, TNFα, and IFNγ in mouse OP models (Yu et al., 2021).

Transplanted stem cells for the improvement of periodontal regeneration have been potentially used to treat periodontal defects. Human periodontal ligament stem cells (hPDLSCs) and jaw bone marrow mesenchymal stem cells (JBMMSCs) are good candidates for periodontal regenerative therapy in the clinic. Osthole (10^−5^ mol/L) increases the ALP activity and upregulates the expression of Col-I, integrin β1, fibronectin, Runx-2, and osteocalcin in hPDLSCs and JBMMSCs. In addition, osthole-mediated hPDLSCs and JBMMSCs produce more new bone in the ectopic *in vivo* transplantation models (Gao et al., 2013). Epigenetic modification plays a critical role in mediating bone metabolism and the pathological development of OP. Specifically, DNA methylation, histone modification, and non-coding RNAs affect the progression of OP (Chen et al., 2023). Inflammatory microenvironment may induce the alterations of epigenetic modification. Inflammatory responses have been reported to cause the defective osteogenic differentiation of P-PDLSCs, and this might be associated with the post-transcriptional modification of histone by acetylation. Osthole (10^−7^ M) can rescue the defective osteogenic differentiation of P-PDLSCs through histone acetylation, as shown by increasing the expression of MOZ, MORF, H3K9ac, and H3K14ac (Sun et al., 2017).

Controversially, one study reports that osthole may inhibit the proliferation and osteogenic differentiation of rat MSCs. Specifically, osthole (6.25, 12.5, and 25 μM) downregulates the expression of Col1a1, Col1a2, OCN, ALP, Runx-2, Osterix, OPN, and OPG and inhibits the mineralized nodule formation and calcium influx in rat MSCs. Mechanically, osthole treatment for 3 h induces negative effects on osteogenesis by suppressing the activities of Wnt/β-catenin and ERK1/2-MAPK signaling pathways (Hu et al., 2016). This discrepancy might be associated with the different drug-interacting periods, cell situations, and culture circumstances.

## 5 Osthole inhibits osteoclastogenesis and osteoclast activity

**FIGURE 4:**
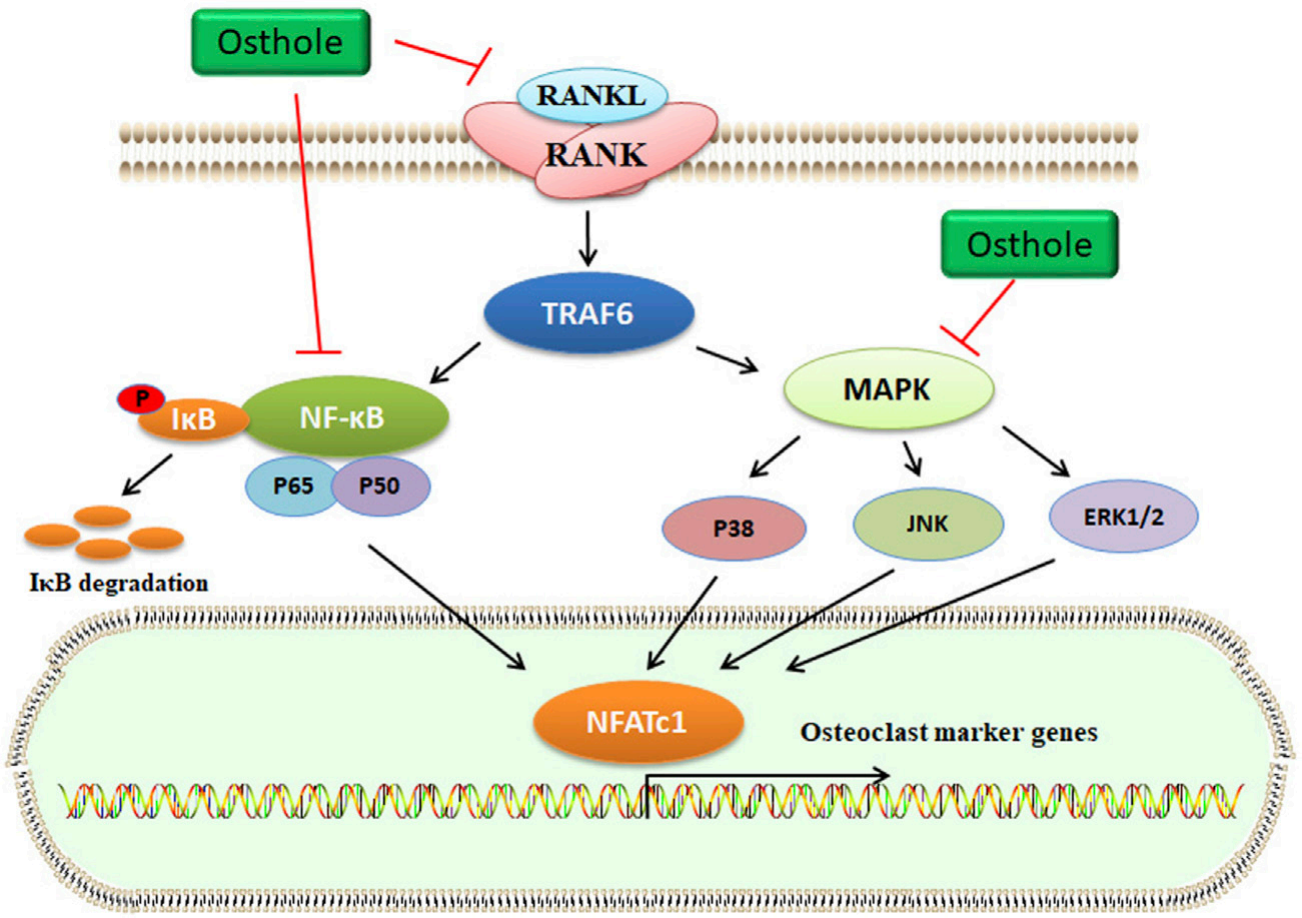
Osthole inactivates the RANKL signaling and blocks osteoclastogenesis. RANKL interacts with RANK, which activates TRAF6 expression. Then, MAPK and NF-κB signaling pathways are activated. Finally, the transcriptional expression of osteoclast marker genes, such as NFATc1, is upregulated. Osthole inhibits the activity of RANKL and suppresses the MAPK and NF-κB signaling pathways, therefore blocking osteoclastogenesis.

Increased osteoclasts activity and excessive bone resorption are associated with osteolytic dysfunctions, such as OP. Osteoclasts derived from the monocyte/macrophage lineage of hematopoietic stem cells are formed via the mediation of some cytokines, such as RANKL. Specifically, RANKL interacts with RANK and then stimulates the accumulation of TNF receptor-associated factor 6 (TRAF6), which activates several signaling pathways, including activator protein-1 (AP-1), NF-κB, and MAPKs (Figure 4). Activated NF-κB signaling upregulates the expression of nuclear factor of activated T-cells cytoplasmic 1 (NFATc1), which plays a crucial role in osteoclast differentiation (Yamashita et al., 2007). The key osteoclast-specific genes, such as tartrate-resistant acid phosphatase (TRAP), β3-integrin, cathepsin K (CTSK), c-Src, and matrix metallopeptidase 9 (MMP-9), have been involved in osteoclast activation (Boyle et al., 2003).

In OVX-induced mouse OP models, osthole downregulates NFATc1 expression, decreases TRAP-positive osteoclast number, and improves bone loss (Table 1). Osthole has been shown to inhibit the expression of RANKL-mediated osteoclastic factors, such as CTSK, TRAP, CAR2, and MMP-9, and suppress osteoclastogenesis of BMMs. However, osthole does not produce inhibitory effects against RANKL-activated p38, ERK, JNK, and NF-κB pathways in BMMs (Zhao et al., 2018). It has been reported that osthole at the dose of 10^−6^–10^−5^ mol/L exhibits inhibitory effects on RANKL-triggered osteoclastogenesis in RAW264.7 cells. In addition, osthole can downregulate the expression of CTSK, c-Src, β3-integrin, MMP-9, NFATc1, and TRAP and attenuate the activity of bone resorption (Ma et al., 2019). Interestingly, NF-κB signaling has become a potential target in treating osteolysis. It has been reported that severe osteosclerosis is observed in NF-κB-knockout mice due to the decreased activity of osteoclasts (Xing et al., 2003). Osthole can inhibit the phosphorylation and degradation of IκBα and inactivate p65 in the cytoplasm in RANKL-treated RAW264.7 cells (Ma et al., 2019) (Table 2).

Another study reports that osthole (5 mg/kg/day for 4 weeks by intraperitoneal injection) may inhibit osteoclast formation and suppress bone loss in aged mice by up-regulating the expression of OPG. However, the inhibitory effects of osthole (100 μM) against osteoclastogenesis are not observed in BMSCs derived from 3-day-old *OPG* ^−/−^ mice (Jin et al., 2021). *β*-Catenin can promote the gene expression of OPG by binding to its promoter, and OPG can interrupt the between RANKL and RANK, thereby decreasing the formation of osteoclasts (Glass et al., 2005). Further study shows that knockout of *β*-catenin may compromise osthole-induced OPG expression in BMSCs derived from 3-day-old *β-catenin* ^*flox/flox*^ mice (Jin et al., 2021) (Table 1 and 2). Due to the poor water solubility and absorbability, osthole has been developed into NSC-osthole, which embeds osthole in N-alkyl-O-sulfonyl chitosan. Similarly, NSC-osthole can inhibit OVX-induced bone loss in rats and suppress RANKL-induced osteoclastogenesis in BMMs by down-regulating the NF-κB signaling pathway (Wang et al., 2020b).

Renal osteodystrophy is characterized by increased bone loss and fragility fracture in patients with chronic kidney disease (CKD). Increased osteoclast formation and enhanced bone resorption and loss can be observed in a 5/6 nephrectomy mouse model (Malluche and Monier-Faugere, 2006). Osthole (5 mg/kg/day for 2 months by intraperitoneal injection) exhibits protective effects against bone loss in 5/6 nephrectomy mice by inhibiting the activity of osteoclasts, as shown by improved biomechanical properties and decreased expression of TRAP, Cathepsin K, and MMP-9. The expression of OPG in the L4 vertebrae of the 5/6 nephrectomy (5/6-NCT) mice is downregulated. However, the expression of RANKL is not affected. Therefore, the OPG/RANKL ratio is decreased in the 5/6-NCT mice, and it promotes osteoclast formation. Osthole treatment can restore OPG/RANKL ratio *in vivo*. Consistently, osthole can upregulate OPG expression and downregulate RANKL expression in mouse calvarial osteoblasts *in vitro* (Li et al., 2016) (Table 1).

Joint arthroplasty is a treatment of severe arthritic joint diseases. Wear debris in the joint space is often embedded into the synovial tissues and stimulates to produce the pro-inflammatory cytokines, such as IL-6, IL-1β, and TNFα, which promote osteoclastogenesis and osteoclast activity and induce bone resorption (Yin et al., 2023). Wear particle-promoted periprosthetic osteolysis has become a major cause of arthroplasty failure. Osthole (10 mg/kg) has been reported to inhibit tricalcium phosphate (TCP) particle-induced release of IL-6 and TNFα, activation of osteoclasts, and promotion of calvarial osteolysis in mice. Specifically, osthole alleviates TCP particle-inhibited osteocalcin and ALP expression and -stimulated TRAP and cathepsin K activity by down-regulating the expression of GRP78 and CHOP in mice (Lv et al., 2016) (Table 1).

## 6 The potential mechanism of osthole in mediating bone remodeling

BMPs, belonging to the transforming growth factor *β* (TGFβ) superfamily, are the potent regulators of osteoblast differentiation and bone generation. BMP-2, the earliest BMP factor, is upregulated to elicit osteogenic signals and drive osteoblast differentiation. For example, BMP-2, by interacting with its receptor BMPRs, may upregulate the expression of osterix, which is associated with osteoblast differentiation. It has been reported that osthole can promote osteoblast differentiation by stimulating BMP-2 signaling (Ming et al., 2011). In chick thiram-induced tibial dyschondroplasia models, the expression of ALP, BMP-2, and Runx-2 is significantly downregulated. Osthole administration can compromise the effects of thiram and promote osteoblast differentiation by up-regulating the expression of BMP-2 and Runx-2 (Waqas et al., 2019). Another study shows that osthole (100 μM) stimulates osteoblast differentiation by increasing the expression of BMP-2, but not BMP-4 and BMP-6, and inducing the phosphorylation and nuclear translocation of Smad1/5/8. Further study exhibits that osthole upregulates the expression of Wnt1, Wnt3a, Wnt4, *β*-catenin, and the target genes axin2, indicating the activation of the Wnt/β-catenin signaling in primary mouse osteoblasts. The induction of osteoblast differentiation by osthole is associated with the activation of *β*-catenin and BMP-2 signaling. And deletion of the *β*-catenin expression by the infection of Ad-Cre into *β-catenin* ^*flox/flox*^ osteoblasts may inhibit osthole-stimulated expression of Runx-2, ALP, BSP, and BMP-2, suggesting that BMP-2 acts as the downstream factor of the *β*-catenin signaling (Tang et al., 2010).

Consistently, osthole stimulates the maturation and differentiation of osteoblast-like cell lines, MG-63 and hFOB, without affecting cell growth, by activating the BMP-2 signaling. Interestingly, the recombinant BMP-2 protein does not promote osthole-induced expression of ALP and osteocalcin, which are the phenotypic markers for the early/mature (Franceschi and Iyer, 1992) and terminal (Jia et al., 2003) differentiation, respectively. Noggin, a BMP-2 inhibitor, exhibits no effects on ALP activity and osteocalcin secretion but abolishes BMP-2-mediated cell differentiation. These suggest that osthole mediates cell differentiation in BMP-2-dependent and -independent manners. Further study indicates that osthole induces the phosphorylation of Smad1/5/8, p38, and ERK1/2, which is essential for osthole-mediated osteoblast differentiation of MG-63 and hFOB cells. The BMP-2 signaling is necessary for osthole-induced activation of p38 signaling but partially for that of ERK1/2 signaling (Kuo et al., 2005). Another study reports that osthole can increase the phosphorylation of cAMP and CREB, the unclear translocation of p-CREB, and the expression of osterix, leading to the enhancement of osteogenesis. PKA acts as the downstream factor of the cAMP/CREB signaling pathway. Treatment with KT5720, an inhibitor of PKA, may markedly suppress osthole-mediated osterix. However, KT5720 does not affect osthole-induced ALP, BMP-2, and Runx-2 expression in MC3T3-E1 cells. Thus, the PKA inhibitor KT5720 may partially suppress the osteogenesis induced by osthole (Zhang et al., 2017).

Inflammation contributes to the activation of osteoclasts and the induction of osteolysis. The osteoclast-released pro-inflammatory cytokines, such as IL-6, IL-1β, and TNFα, may, in turn, promote osteoclastogenesis (Madel et al., 2019). Macrophages have two different phenotypes, such as pro-inflammatory M1 type and anti-inflammatory M2 type. The transition between M1 and M2 is dependent on the bone immune microenvironment. An M1 polarization of macrophages has been associated with alveolar bone resorption (Zhou et al., 2019). It has been reported that osthole can suppress lipopolysaccharide (LPS)-induced M1 macrophage polarization of BMMs, inflammatory responses, and osteolysis. Specifically, osthole at the dose of 25 μM may stimulate M2 polarization of macrophages, suppress the expression of IL-6, iNOS, CD86, and CCR7, and decrease LPS- and RANKL-induced generation of ROS, inhibiting osteoclastogenesis and osteoclast-specific gene expression by down-regulating the p38-MAPK/NF-κB signaling pathway (Wang et al., 2023a).

## 7 The pharmacokinetic profiles of osthole and its metabolites protect against OP development

The pharmacokinetics of osthole in rat plasma after a dose of 10 mg/kg by intravenous administration has been studied using the high-performance liquid chromatography (HPLC) method. The values of mean residence time (MRT) and half-life time (*T*_1/2_) are 48.41 min and 41.13 min, respectively (Tsai et al., 1996). Another study shows that the values of *C*_max_, AUC, and *T*_1/2_ are 0.7271 ± 0.00206 μg/mL, 5.3529 ± 0.0706 μg h/mL, and 3.6998 ± 0.0755 h, respectively, after oral administration of 130 mg/kg osthole in rats. In addition, a two-compartment open pharmacokinetic model indicates that osthole can be absorbed rapidly and effectively and eliminated slowly (Shi et al., 2013). After oral administration of free osthole (20 mg/kg) in healthy male SD rats, the *C*_max_, *T*_max_, AUC, and *T*_*1/2*_ of osthole are 366 ± 89 ng/mL, 0.61 ± 0.09 h, 780 ± 585 ng·h/mL, and 4.94 ± 1.84 h, respectively (Zhou et al., 2011).

Osthole is one of the bioactive compounds in the Bushen Yizhi (BSYZ) formula. After oral administration of osthole (75 mg/kg) and BSYZ (15 mg/kg osthole) extract in male SD rats, the bioavailability of osthole is significantly enhanced due to the synergistic effects of the bioactive compounds in the BSYZ formula. Specifically, the *C*_max_, AUC_0→t_, and AUC_0→∞_ of osthole in BSYZ are 0.313 ± 0.043 ng/mL, 2.42 ± 0.20 μg h/mL, and 2.62 ± 0.30 μg h/mL, respectively. Comparably, these values of pure osthole are 0.114 ± 0.027 ng/mL, 0.63 ± 0.12 μg h/mL, and 0.78 ± 0.14 μg h/mL, respectively (Zhang et al., 2014). It has been reported that CYP3A4 is the enzyme metabolizing osthole. It is interesting that the other bioactive compounds, such as Schisantherin B, in the BSYZ formula may inhibit the activity of CYP3A4, significantly enhancing the bioavailability of osthole (Liu et al., 2022).

It has been demonstrated that osthole can increase the expression of CYP7A (Du et al., 2011) and CYP11B1 (Pan et al., 2015) and decrease the expression of CYP2E1 (Zhang et al., 2011). CYP3A accounts for an appropriate 30% of the total CYP450 enzyme in the liver, and CYP3A metabolizes about 50% of the marketed drugs. Osthole has been reported to decrease the mRNA expression of CYP3A1 but not CYP3A2. However, osthole inhibits the protein expression of CYP3A1 and CYP3A2 (Huang et al., 2020). Another study reports that osthole can suppress the activity of human CYP2C11 and CYP2C9 and affect the pharmacokinetics of indomethacin *in vivo* (He et al., 2020). These indicate that osthole may induce toxic adverse effects and affect the metabolism of other drugs by negatively or positively mediating the expression of some CYP450 enzymes. In the acute toxicity testing of osthole for goldfish, the median lethal dose (LD_50_) value is 6.749 mg/L (Wang et al., 2008). However, the information about the LD_50_ values of osthole for other animals is limited.

Borneol has been reported to inhibit CYP450 enzymes (Jinno et al., 2014) and affect p-glycoprotein activity (He et al., 2011). Interestingly, borneol may increase the *C*_max_, AUC_0→t_, and AUC_0→∞_ of osthole in male SD rats, significantly enhancing the bioavailability of osthole (Luo et al., 2017). To improve the bioavailability and tissue targeting, transferrin-modified osthole liposomes (TF-Ost-Lip) have been developed. After intravenous administration of 10 mg/kg TF-Ost-Lip or osthole in SD rats, the AUC_0→48 h_ value of 2.448 ± 0.861 or 0.119 ± 0.027, respectively (Kong et al., 2020). Similarly, osthole is formulated with the liquid self-microemulsifying drug delivery system (L-SMEDDS) and further with the solid SMEDDS (S-SMEDDS), which increases the bioavailability of osthole by 205%, as indicated by a prolonged time of *T*_max_ and MRT (Sun et al., 2018). Osthole can be developed to be osthole-polybutyl cyanoacrylate nanoparticles (Osthole-PBCA NPs), which increases the value of AUC 3.3 times, significantly enhancing the bioavailability (Zheng et al., 2022).

Osthole can be absorbed into the systemic circulation, and the peak concentration of osthole after oral administration can be reached rapidly in rats. The absorbed osthole can be metabolized rapidly and extensively. One study reports that 7-demethylation and 8-dehydrogenation in osthole are the major metabolic reactions in phase I metabolism using the incubation of microsome separated from rats (RLM) (Yuan et al., 2009). Another study suggests that using RLM is not an effective strategy to mimic the reactions in phase I metabolism *in vivo* because of the difference between *in vivo* and *in vitro*. Ten phase-I and three phase-II metabolites have been detected in the urine after oral administration of 40 mg/kg osthole. Consistently, hydroxylation, demethylation, and hydrogenation are the major metabolic reactions in phase I metabolism. Glucuronidation is the major reaction in the phase-II metabolism in rats, while sulfates are not detected in bile, urine, or feces using HPLC-DAD-MS/MS system (Lv et al., 2012a). The comparative metabolite profiles of osthole in OP and healthy rats show that 36 phase-I and phase-II metabolites are identified in the plasma of OP rats, which are treated with 40 mg/kg for 12 weeks by oral administration. The hydrolysis products of osthole are detected in OP rats but not in healthy rats. It is interesting to find the sulfate and glucuronide conjugations of osthole in OP rats using LC-QTOF/MS system (Wang et al., 2018).

The serum metabolomics of OVX-induced OP rats treated by osthole has been analyzed by the UPLC/Q-TOF-MS system. 19 endogenous metabolites associated with 13 metabolic pathways have been detected in OVX-induced OP rats, which are treated with osthole (400 mg/kg/day for 8 weeks) after model duplication. Twenty-eight metabolites have been highlighted as the potential biomarkers of OVX-induced OP in rats. Interestingly, the content of lysine, arginine, ornithine, and tryptophan in OP rats is enhanced, while that of glutamine is declined. In addition, the metabolism of unsaturated fatty acids, lipids, amino acids, carbohydrates, bile acids, purines, and the TCA cycle is involved in the pathogenesis of OP (Si et al., 2020).

Biotransformation, characterized by the advantages of simple operation procedures, mild reaction conditions, and high regio- and stereo-selectivity, provides an economical technology for the analog preparation of effective compounds. The biotransformation of osthole by *Alternaria longipes* (Xin et al., 2013), *Mucor spinosus* (Lv et al., 2012b), and *Mucor circinelloides* (He et al., 2023) has been investigated. Various modifications, such as hydroxylation, glycosylation, dehydrogenation, demethylation, and glucuronidation, have been prepared to modulate the physicochemical properties, such as structural stability and water solubility. It has been reported that glycosylation, dehydrogenation, and demethylation can decrease the biological effects of osthole on the proliferation of MC3T3-E1 cells. However, the hydroxylation at C-4′ position can increase the water solubility and enhance the proliferative activity of MC3T3-E1 cells (Lv et al., 2012b). Inconsistently, the glycosylation at the C-3 position and the hydroxylation at the C-4′ or C-5’ position may increase the water solubility, enhance the proliferative activity of MC3T3-E1 cells, and improve the anti-OP effects (He et al., 2023). It is important to note that the metabolites of osthole might be responsible for the pharmacological activities. The transformation mediated by intestinal fungi may provide a novel strategy to explore the efficacy of osthole economically and safely.

## 8 Critical discussion

Over the past 20 years, various drugs for treating OP have appeared. They include bisphosphonates, recombinant human PTH, denosumab, SERMs, Vitamin D analogs, and ipriflavone (Ponnapakkam et al., 2014). However, long-term administration of these drugs may be associated with untoward effects, such as the development of breast cancer, ovarian cancer, heart attack, thrombogenesis, and jaw osteonecrosis (Tile and Cheung, 2020). Natural medicinal plants have shown efficacy for thousands of years with fewer side effects. Natural compounds are the bioactive components responsible for the pharmacological effects of traditional medicinal plants. Osthole has multiple pharmacological activities, such as anti-OP. Osthole promotes osteoblast-mediated new bone form, suppresses osteoclast-regulated bone resorption, and therefore maintains bone remodeling (Tang et al., 2010; Ma et al., 2019).

The osteogenesis of BMSCs is critical for bone growth, fracture healing, and osseointegration (Huo and Yue, 2020). The early mediators of BMSC osteogenesis involve endocrine hormones, BMPs, Wnt/β-catenin, and hedgehog signaling pathways (D'Alimonte et al., 2013). The expression of RUNX-2 and osterix 1 is crucial for the osteogenesis of BMSCs. The BMPs signaling is essential for the proper function of osteoblasts, which secrete the matrix for bone formation. Conditional double knockout of BMP2/BMP4 in mice results in the abnormality of chondrocyte differentiation and hypertrophy within the growth plate during endochondral bone formation (Shu et al., 2011). Smads transduce the signal of the BMP pathway to the nucleus and mediate the transcriptional expression of target genes involved in osteoblastic/chondrogenic differentiation (Xiao et al., 2021). Activation of the BMP signaling contributes to the osteochondrial lineage differentiation of human MSCs (Chan et al., 2022). Osthole drives the osteoinductive activity of BMSC by activating the BMP-2/Smad1/5/8 signaling. However, the specific targets of osthole are still unknown. TGFβ promotes ECM deposition and collagen distribution and induces the tenogenic differentiation of human adipose-derived stem cells (Scioli et al., 2019; Wang et al., 2020a). BMP-2 and TGFβ promote the osteogenic and tenogenic differentiation of ligament-derived stem cells (LDSCs) (Wei et al., 2023). However, TGF-β and BMP signaling pathways are antagonized by different components (Loh et al., 2023). One study reports that osthole can reduce the expression of TGFβ, Smad2, and Smad3 and block TGFβ-induced Smad2/3 phosphorylation and nuclear translocation (Liu et al., 2018; Yue et al., 2020). Similarly, TGFβ suppresses phosphate-induced osteogenesis by inhibiting the BMP and Wnt/β-catenin signaling pathways (Guerrero et al., 2014). The effects of osthole on TGFβ1-mediated bone metabolism are still unclear.

BMP-2 cooperates with the Wnt/β-catenin signaling to mediate osteogenic differentiation and bone formation. The cross-talk between BMP-2 and the Wnt/β-catenin signaling in bone has been discussed (Marini et al., 2023). BMP-2 acts as a downstream factor of the Wnt/β-catenin signaling. Whether osthole stimulates BMP-2 expression by activating the Wnt/β-catenin signaling remains to be further investigated. The interaction between osthole and *β*-catenin has not been reported. GSK-3β negatively regulates the Wnt/β-catenin signaling. It has been reported that osthole can reduce the phosphorylation of GSK-3β and inactivate its activity (Huang et al., 2023). The underlying mechanism of osthole in regulating the Wnt/β-catenin signaling in bone needs further study. In addition, the metabolites of osthole may exhibit pharmacological effects on osteogenesis and osteoclastogenesis. It is necessary to investigate the potential roles of these metabolites in bone remodeling.

Osthole may inhibit the production of pro-inflammatory cytokines and suppress the NF-κB and MAPK signaling pathways in D-galactose-treated liver tissues (Ma et al., 2023). Similarly, osthole reduces LPS-induced inflammatory responses by inhibiting the NF-κB signaling pathway in Caco-2 cells (Kordulewska et al., 2020). Osthole also inhibits the expression of COX-2, MMP-13, and RUNX-2 by suppressing the NF-κB and HIF-2α signaling pathways in osteoarthritic chondrocytes (Chern et al., 2020). Controversially, osthole does not affect the NF-κB and MAPK signaling pathways in RANKL-treated BMMs (Zhao et al., 2018). This discrepancy might be associated with the different cell lines and cell microenvironments. However, the molecular mechanism of osthole in inhibiting the NF-κB and MAPK signaling pathways in bone is still unknown. Furthermore, how osthole inhibits RANKL-stimulated osteoclastogenesis still needs further investigation.

The structure-activity relationship of osthole in mediating bone remodeling is still unknown. Prenylation may enhance the affinity of compounds to cell membranes, even the interaction with the potential target proteins. For example, prenylation significantly enhances the uptake of 8-prenyl quercetin (Mukai et al., 2013). Prenylation also enhances the estrogenic effects of naringenin and genistein (Kretzschmar et al., 2010). It has been reported that the isopentenyl group in psoralidin strongly contributes to the inductive activity of the osteogenic differentiation and mineralization of MC3T3-E1 cells (Xu et al., 2022). Coumestrol, due to the absence of an isopentenyl group in psoralidin, has comparably low activity on osteoprotection (Zhai et al., 2017). The roles of the isopentenyl group in osthole in mediating bone remodeling and protecting against OP development need further study.

## 9 Conclusion

Due to the uncertain effects of bisphosphonates on fragility fractures, alternative medicines for OP treatment have been developed. Osthole has demonstrated a therapeutic effect against the pathological development of OP. More specifically, osthole increases osteoblast-related bone formation by promoting osteogenic differentiation and suppresses osteoclast-related bone resorption by inhibiting osteoclastogenesis and osteoclast activity. The potential mechanism of osthole in mediating bone remodeling might be associated with the activation of Wnt/β-catenin and BMP-2/Smad1/5/8 signaling pathways and the suppression of RANKL pathway. However, the mechanism of how osthole interacts with the BMP-2, Wnt/β-catenin, and RANKL signaling pathways is still unclear. The potential targets of osthole are unclear, and the structure-activity relationship of osthole needs to be elucidated. It should be noted that the biotransformation of osthole may produce effective metabolites, which are effective in protecting against OP development. Whether are the parent forms, the metabolites, or both responsible for the protective activity against OP? Whether the experimental data from the study in osthole-treated cells are valid to explain the pharmacological effects of osthole in animals should be careful verified. The pharmacokinetic profiles of osthole still need further investigation. Due to the poor water solubility, osthole can be therapeutically developed by preparing an effective delivery system, although some achievements have been made. Till now, most studies on the biological effects of osthole are performed in cultured cells and animal models. The pharmacological activity of osthole, particularly in protecting against OP, should be investigated in human beings for clinical trials.
